# Supplementary figures and images for: Whole genome sequence analysis showing unique SARS-CoV-2 lineages of B.1.524 and AU.2 in Malaysia
Source: PLoS One. 2022 Feb 25;17(2):e0263678. doi: 10.1371/journal.pone.0263678 (PMC8880882; doi:10.1371/journal.pone.0263678)

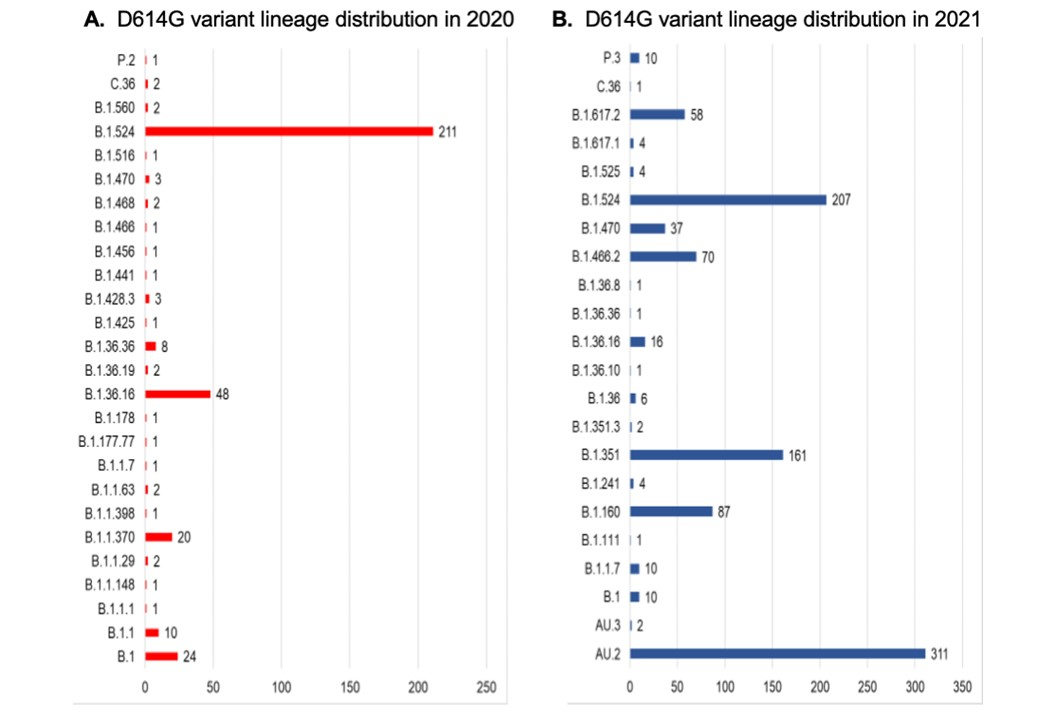

Supplement: S1 Fig — A. The distribution of lineages from March to December, 2020. B. The distribution of lineages from January to July, 2020. (TIFF) [file pone.0263678.s001.tiff]
