# Supplementary material for: Whole genome sequence analysis showing unique SARS-CoV-2 lineages of B.1.524 and AU.2 in Malaysia
Source: PLoS One. 2022 Feb 25;17(2):e0263678. doi: 10.1371/journal.pone.0263678 (PMC8880882; doi:10.1371/journal.pone.0263678)
Supplement: S2 Table — (PDF) [file pone.0263678.s005.pdf]

S2 Table 2: Summary of date and D614G variant virus strains from each lineage first detected in Malaysia.

Clade	Lineage	PANGO lineage description	Date	Genomes
GH	AU.2	Alias of B.1.466.2.2, Malaysia lineage	January 3, 2021	UNIMAS-B195/2021, UNIMAS-B187/2021, UNIMAS-B188/2021
	AU.3	Alias of B.1.466.2.3, predominantly Australia with some Papua New Guinea and Singapore sequences	March 25, 2021	UNIMAS-16844/2021
	B.1	A large European lineage the origin of which roughly corresponds to the Northern Italian outbreak early in 2020.	April 7, 2020	MGI-G873/2020
	B.1.111	South/ Central American lineage (mostly Trinidad and Colombia, a small cluster from Norwich)	February 21,2021	UNIMAS-16404/2021
	B.1.160	Large European lineage, sequences from BeNeLuX, Denmark, Switzerland, Hungary and UK.	February 3, 2021	UNIMAS-B186/2021
	B.1.351	Lineage of concern detected in South Africa	February 1,2021	IMR_WC75452/2021
	B.1.351.3	Sublineage of B.1.351 predominantly in Bangladesh and Singapore with exports elsewhere	May 4,2021	IMR_DOB130393/2021, IMR_DOB140382/2021
	B.1.36.16	SE Asia	October 16,2020	IMR-WI194/2020,IMR-W195I/2020
	B.1.36.19	Oceana	October 28,2020	UNIMAS-15723/2020, UNIMAS-15729/2020
	B.1.36.36	Canada	March 26,2020	NPHL_14135/2020
	B.1.36.8	Prodominantly Indian lineage with global records (inc. Canada)	January 17,2021	IMR_WC39298/2021
	B.1.428.3	UAE, Jordan	October 27,2020	MGI-DNALAB12/2020
	B.1.441	Global lineage (inc. merge of B.1.274)	December 24,2020	UNIMAS-15834/2020
	B.1.456	SE Asian (reasigned)	October 8,2020	UNIMAS-15694/2020
	B.1.466	Global lineage containing two new sublineages	June 9,2020	NPHL_98435/2020
	B.1.466.2	Indonesia	January 10,2021	UNIMAS-15877/2021
	B.1.468	Indonesia/Sinagpore. Seq names missassigned in original file	April 2,2020	IIUM91/2020
	B.1.470	Indonesia/Singapore	October 14,2020	UNIMAS-15695/2020
	B.1.516	USA (AZ and NM)	April 14,2020	NPHL_37335/2020
G	B.1	A large European lineage the origin of which roughly corresponds to the Northern Italian outbreak early in 2020.	March 31,2020	UNIMAS-0099/2020
	B.1.1.148	US lineage	November 6,2020	UNIMAS-B072/2020
	B.1.178	Democratic Republic of the Congo lineage	April 10,2020	IMR_WC12897/2020
	B.1.241	USA lineage	March 17,2021	IMR_WC139765/2021
	B.1.36	Global many subliniages	May 4,2021	IMR_86285/2021, IMR_180646/2021, IMR_25737/2021, IMR_86009/2021, IMR_85793/2021
	B.1.36.10	Jordan/Saudi Arbia (single recent record from USA)	January 4,2021	8827/2021

S2 Table 2 -Continue

Clade	Lineage	PANGO lineage description	Date	Genomes
G	B.1.36.36	Canada	March 26,2020	NPHL_14135/2020
	B.1.425	USA lineage	April 12,2020	8451/2020
	B.1.524	Malaysian lineage	September 1,2020	IMR-CV138548/2020, IMR-CV138551/2020, IMR-CV138687/2020, IMR-CV138815/2020, IMR-CV139140/2020, IMR-CV139154/2020, IMR-CV139158/2020, IMR-CV139176/2020, IMR-CV139206/2020, IMR-CV1140107/2020, IMR-CV140153/2020, IMR-CV140273/2020, IMR-CV140277/2020, IMR-CV140278/2020, IMR-CV140314/2020, IMR-CV140338/2020, IMRCV140352/2020, IMR_CV140342/2020, IMR_CV140329/2020, IMR-CV136859/2020, IMR-CV138522/2020, IMR-CV138523/2020
	B.1.525	International lineage with E484K, del69-70 among other defining mutations.	January 31,2021	IMR_WC74211/2021, IMR_WC74210/2021
	B.1.560	India	April 13,2020	UNIMAS-M0710/2020
	B.1.617.1	Predominantly India lineage with 484Q, pango-designation issue #49	April 10,2021	IMR_WC185230/2021
	B.1.617.2	Predominantly India lineage with several spike mutations, pango-designation issue #49	April 28,2021	IMR_SB68524/2021
GR	B.1.1	European lineage with 3 clear SNPs \`28881GA\`,\`28882GA\`,\`28883GC\`	March 21,2020	0121/2020
	B.1.1.1	England	May 10, 2020	IMR_WC55122/2020
	B.1.1.29	Wales lineage, formerly temporarily assigned B.1.1.439 due to an error	March 22,2020	0309/2020
	B.1.1.370	England, USA, Russia, Germany	July 27,2020	IMR-WI085/2020, IMR-WI080/2020
	B.1.1.398	USA/ Indonesian lineage	December 7,2020	IMR_WC317894/2020
	B.1.1.63	Hong Kong lineage	July 25,2020	IMR-WI109/2020, IMR-WC124489/2020
	C.36	Alias of B.1.1.1.36, Egypt mainly and other countries	May 29,2020	IMR_WC90685/2020
	P.2	Alias of B.1.1.28.2, Brazilian lineage	December 15,2020	IMR_WC348412/2020
	P.3	Alias of B.1.1.28.3, Lineage predominantly in the Philippines with spike mutations E484K, N501Y, P681H, 141-143del	March 23,2021	UNIMAS-16809/2021

S2 Table 2 -Continue

Clade	Lineage	PANGO lineage description	Date	Genomes
GRY	B.1.1.7	UK lineage of concern, associated with the N501Y mutation	December 28,2020	IMR_WC378731/2020
GV	B.1.177.77	Benelux + Denmark	October 1,2020	IMR-WC194446/2020
O	B.1	A large European lineage the origin of which roughly corresponds to the Northern Italian outbreak early in 2020.	April 2,20202	UNIMAS-0298/2020
	B.1.1	European lineage with 3 clear SNPs \`28881GA\`,\`28882GA\`,\`28883GC\`	March 31,2020	UNIMAS-0136/2020
	B.1.560	India	April 13,2020	UNIMAS-1741/2020

- The data presented on this table were based on the metadata of patient status downloaded to GISAID, July 19, 2021.Both Malaysian lineage B.1.524 and AU.2 were highlighted in grey. The linage description listed as described by PANGO (https://cov-lineages.org/).
